# Supplementary material for: The Role of Insulin-like Growth Factor Binding Protein (IGFBP)-2 in DNA Repair and Chemoresistance in Breast Cancer Cells
Source: Cancers (Basel). 2024 May 31;16(11):2113. doi: 10.3390/cancers16112113 (PMC11171178; doi:10.3390/cancers16112113)
Supplement: Supplementary file 1 [file cancers-16-02113-s001.zip › cancers-2993826-Figure S1.pdf]

Supplementary Figure S1

A.

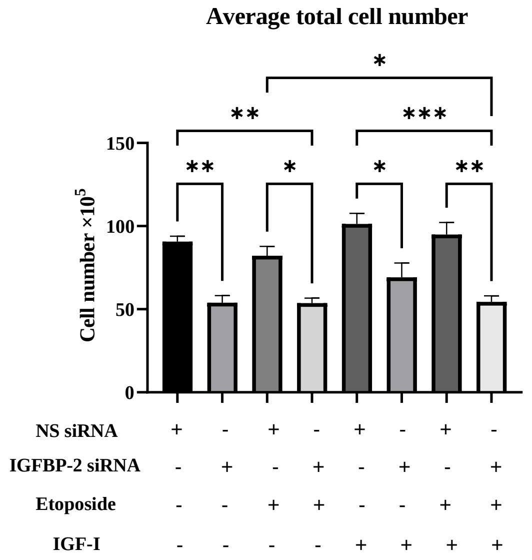

B.

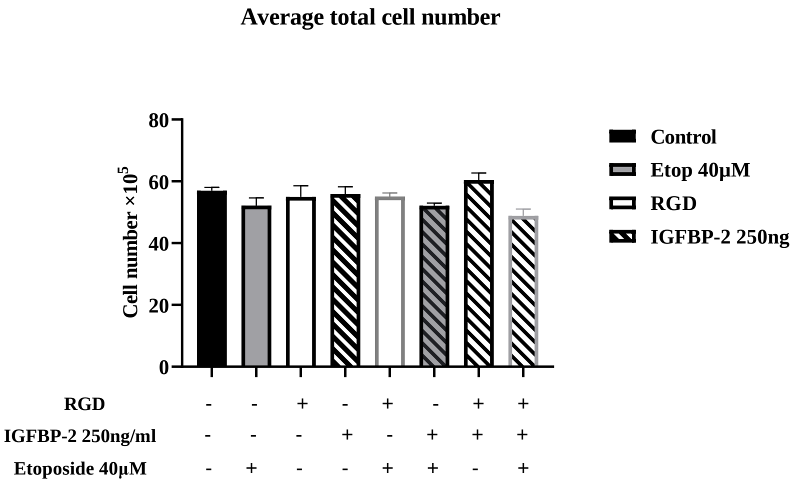

C.

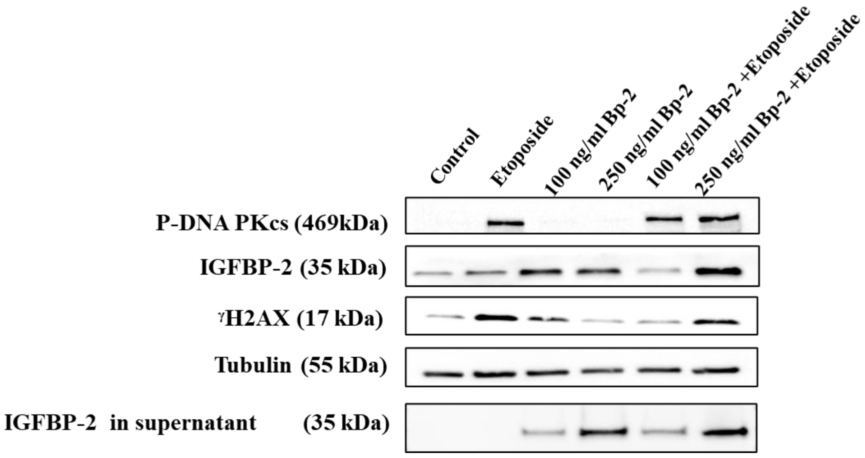

Supplementary Figure S1 – (A). IGFBP-2 silencing in MCF-7 cells with or without IGF-I and etoposide. (**B)**. MDA-MB-231 cells dosed with recombinant IGFBP-2 with or without etoposide in the presence or absence of RGD. Graphs show the mean+/- se changes in total cell number with each experiment repeated three times in triplicate. **(C)**. MDA-MB-231 cells dosed with recombinant IGFBP-2 (100 and 250 ng/ml) with or without etoposide 40 μM. Representative western blot analysis of cell lysates and supernatants of blots repeated three times.
