# Supplementary material for: The Role of Insulin-like Growth Factor Binding Protein (IGFBP)-2 in DNA Repair and Chemoresistance in Breast Cancer Cells
Source: Cancers (Basel). 2024 May 31;16(11):2113. doi: 10.3390/cancers16112113 (PMC11171178; doi:10.3390/cancers16112113)

A novel role for insulin-like growth factor binding protein (IGFBP)-2 in DNA repair in breast cancer cells.

Original blot

Figure 1B page 4

Original blot in MCF-7 cells

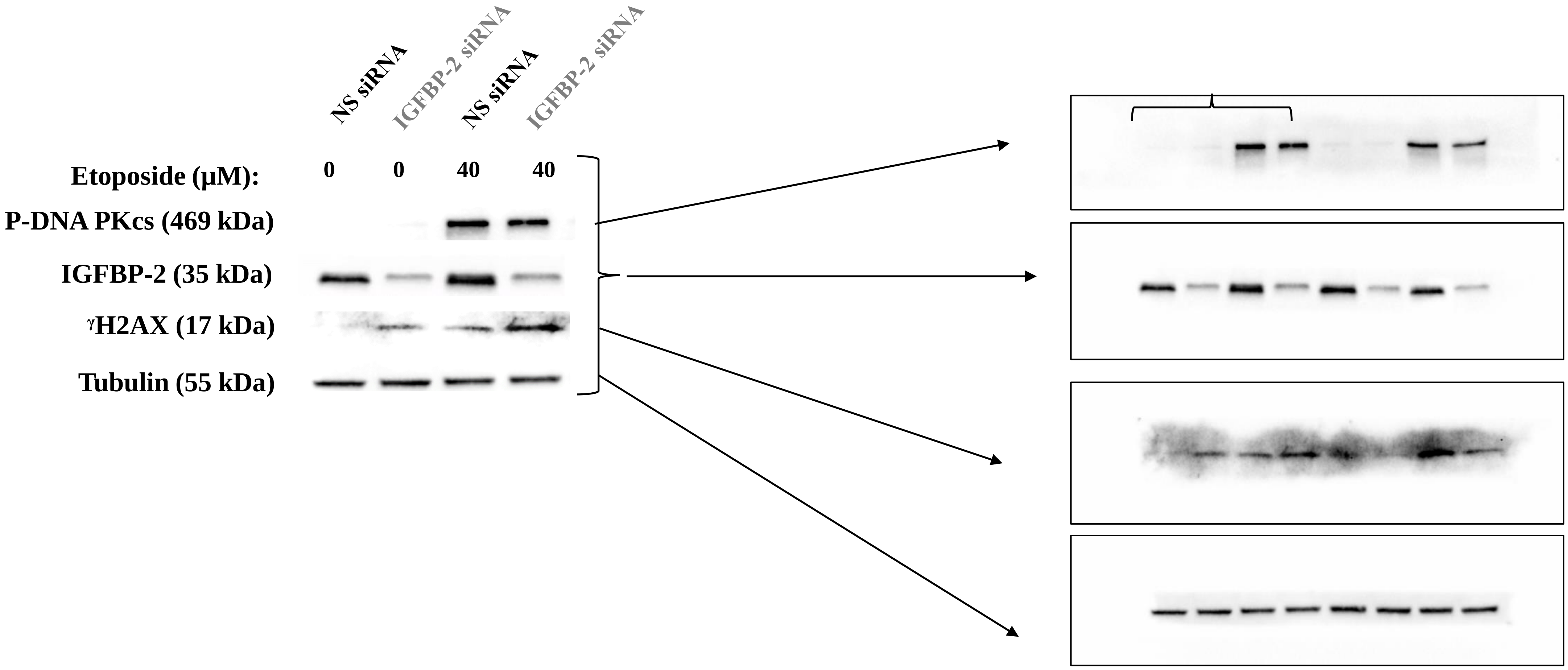

Figure 1E page 4

Original blot in T47-D cells

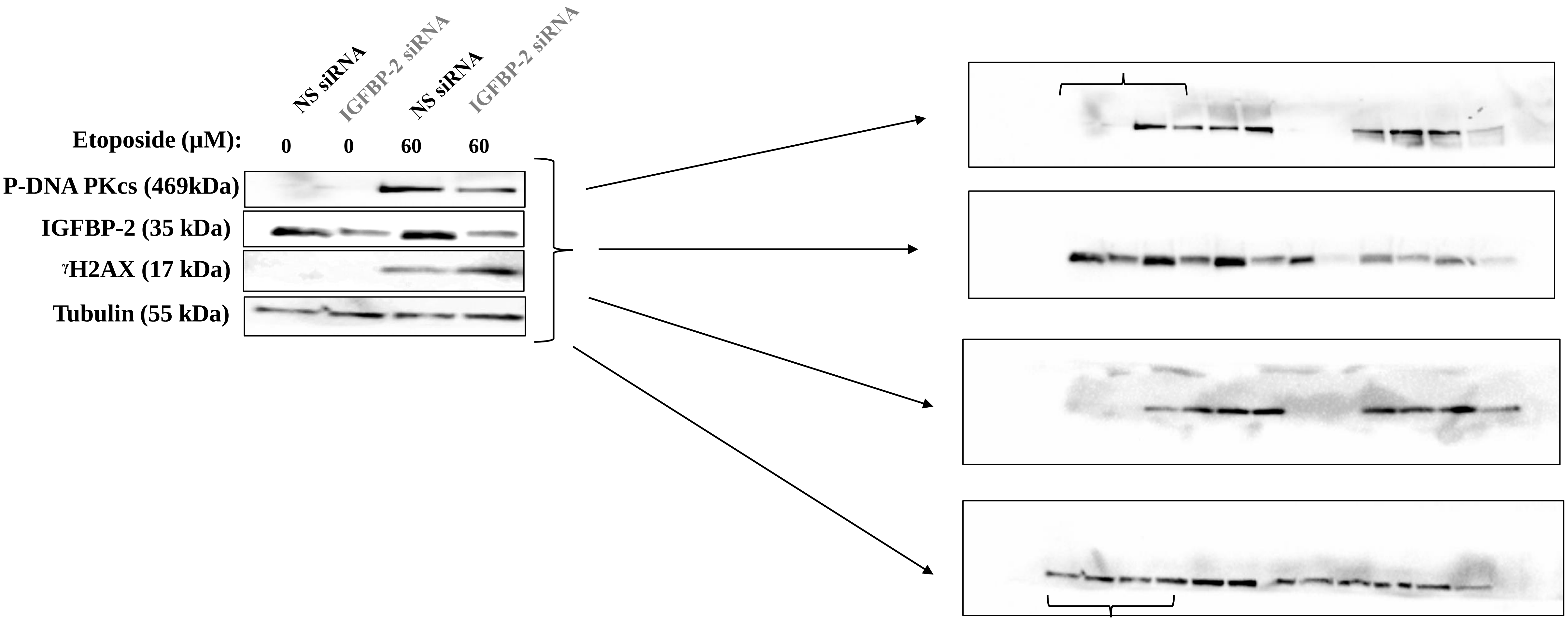

Figure 3B page 6

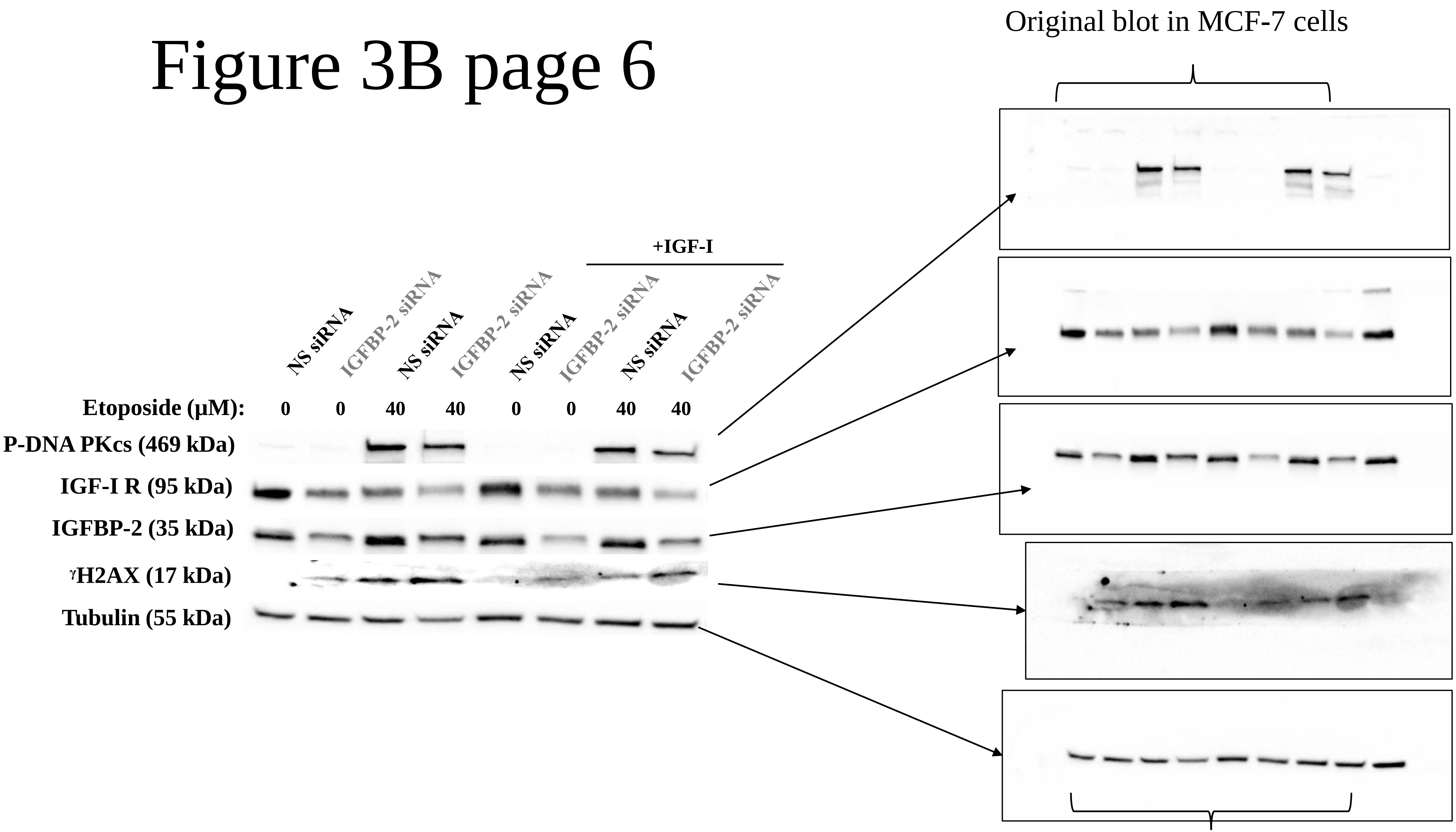

Figure 3D page 7

Original blot in MCF-7 cells

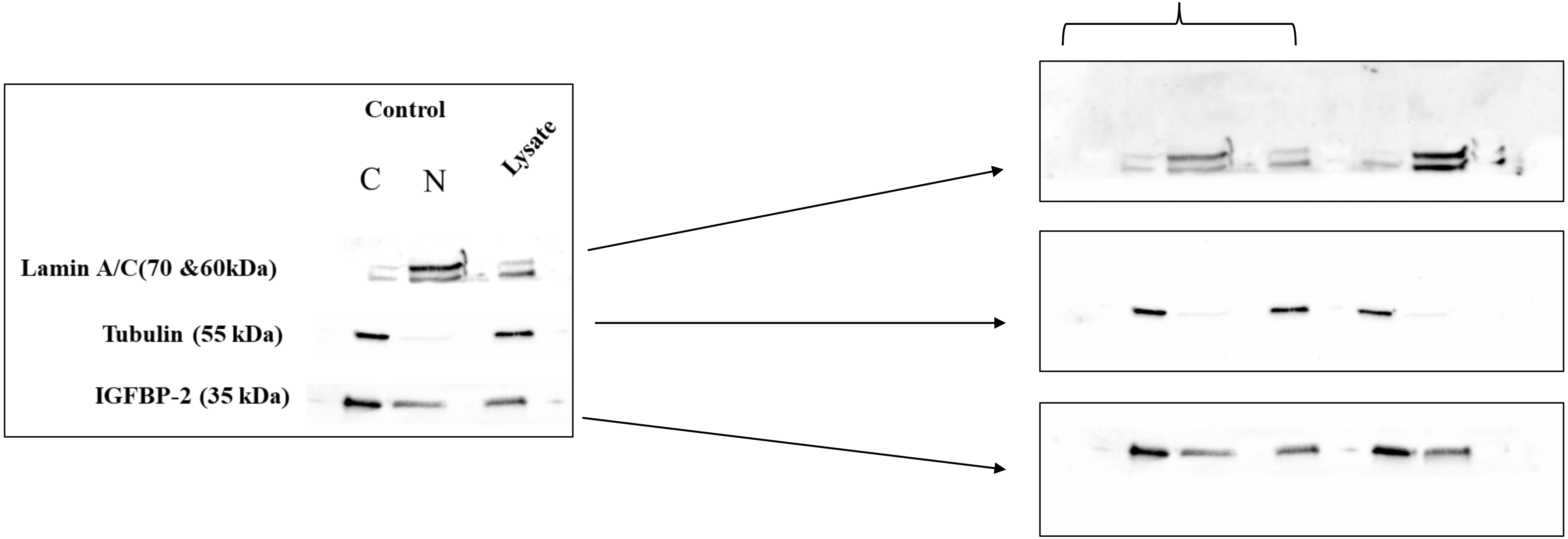

Figure 4B page 10

Original blot in MDA-MB-231 cells

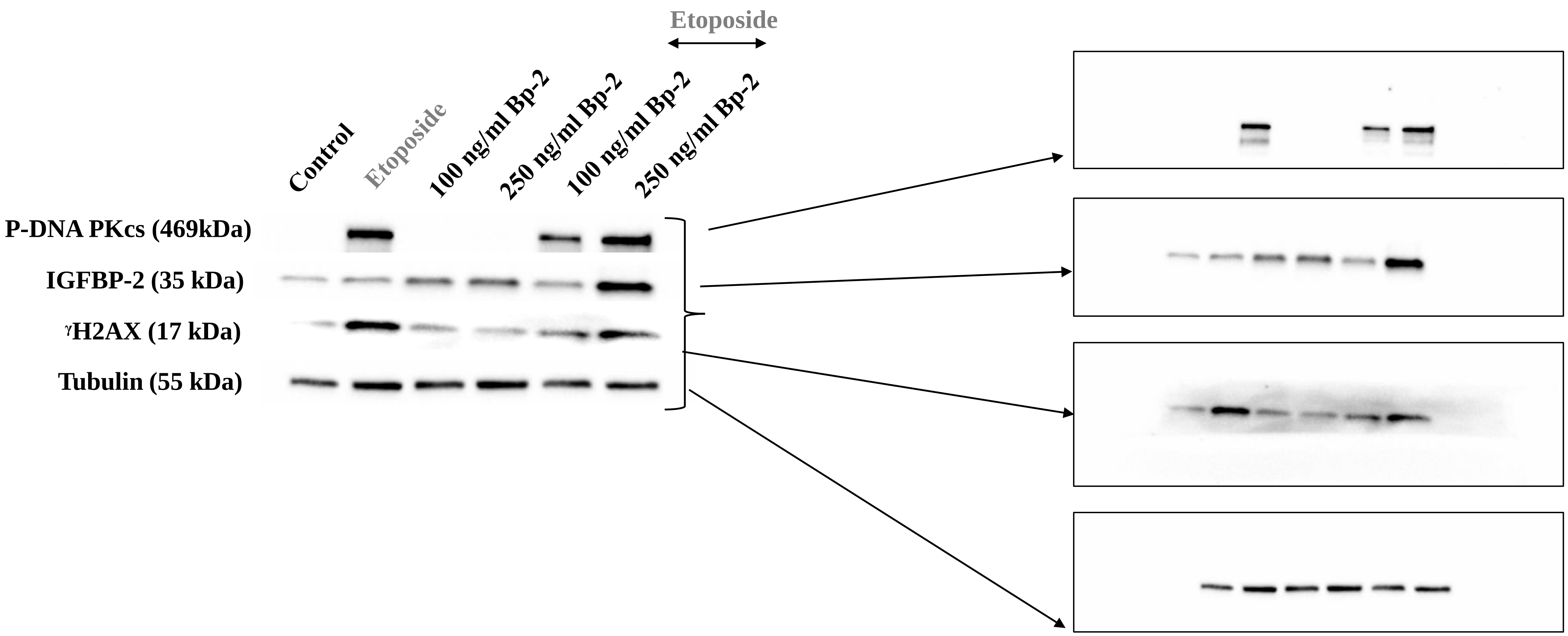

Figure 5B page 11

Original blot in MDA-MB-231 cells

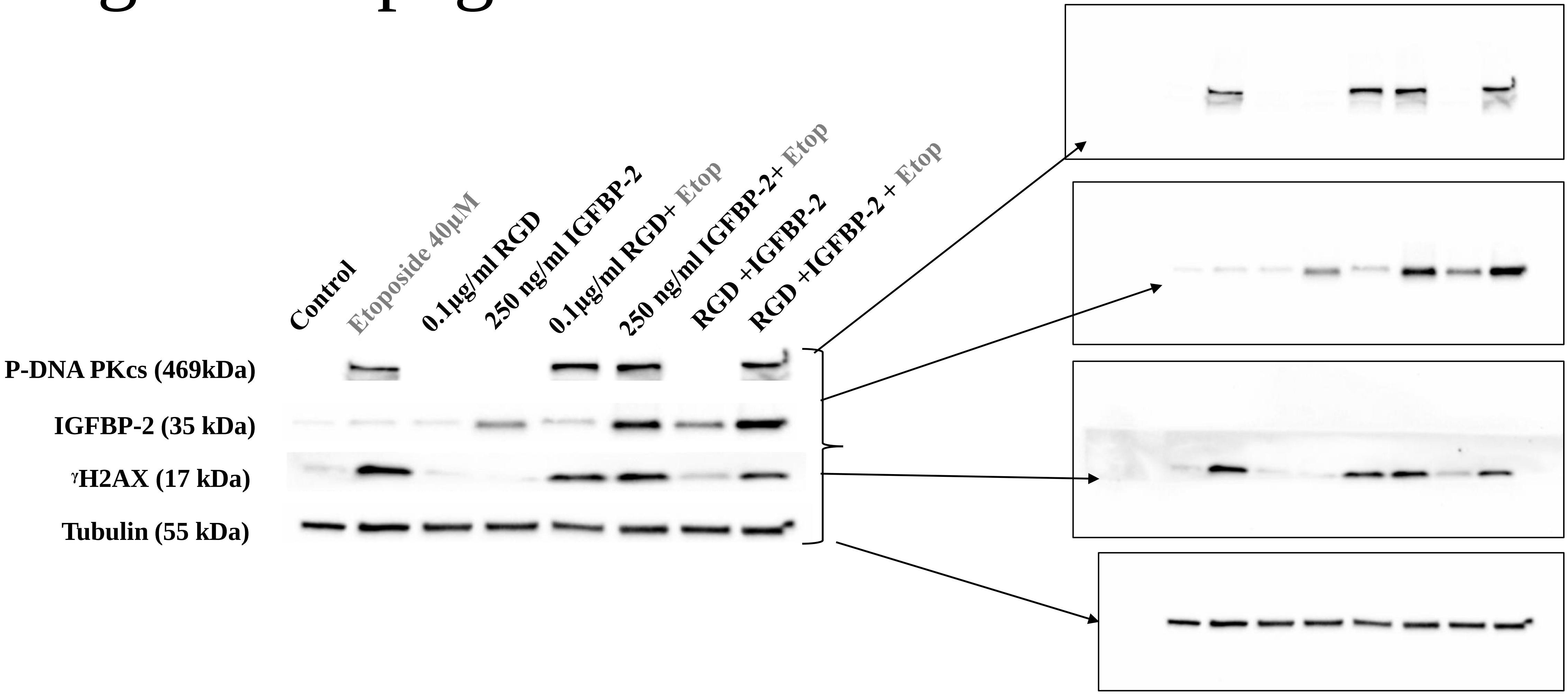

Supplement: Supplementary file 1 [file cancers-16-02113-s001.zip › cancers-2993826-File S1.pdf]
